# Distinct mandibular premolar crown morphology in *Homo naledi* and its implications for the evolution of *Homo* species in southern Africa

**DOI:** 10.1038/s41598-020-69993-x

**Published:** 2020-08-06

**Authors:** Thomas W. Davies, Lucas K. Delezene, Philipp Gunz, Jean-Jacques Hublin, Lee R. Berger, Agness Gidna, Matthew M. Skinner

**Affiliations:** 1 Department of Human Evolution, Max Planck Institute for Evolutionary Anthropology, Deutscher Platz 6, 04103 Leipzig, Germany; 2 School of Anthropology and Conservation, University of Kent, Canterbury, CT2 7NZ UK; 3 Department of Anthropology, University of Arkansas, Fayetteville, AR 72701 USA; 4 Evolutionary Studies Institute, University of the Witwatersrand, 1 Jan Smuts Avenue, Braamfontein, Johannesburg, 2000 South Africa; 5 Collège de France, 11 Place Marcellin Berthelot, 75005 Paris, France; 6 Paleontology Unit, National Museum of Tanzania, Dar es Salaam, Tanzania

**Keywords:** Biological anthropology, Palaeontology

## Abstract

*Homo naledi* displays a combination of features across the skeleton not found in any other hominin taxon, which has hindered attempts to determine its placement within the hominin clade. Using geometric morphometrics, we assess the morphology of the mandibular premolars of the species at the enamel-dentine junction (EDJ). Comparing with specimens of *Paranthropus, Australopithecus* and *Homo* (*n* = 97), we find that the *H. naledi* premolars from the Dinaledi chamber consistently display a suite of traits (e.g., tall crown, well-developed P_3_ and P_4_ metaconid, strongly developed P_3_ mesial marginal ridge, and a P_3_ > P_4_ size relationship) that distinguish them from known hominin groups. Premolars from a second locality, the Lesedi Chamber, are consistent with this morphology. We also find that two specimens from South Africa, SK 96 (usually attributed to *Paranthropus*) and Stw 80 (*Homo* sp.), show similarities to the species, and we discuss a potential evolutionary link between *H. naledi* and hominins from Sterkfontein and Swartkrans.

## Introduction

*Homo naledi* is a hominin species first described in 2015 based on remains from the Dinaledi Chamber in the Rising Star cave system in South Africa^1^, and subsequently from a second chamber in the cave, the Lesedi Chamber^2^. The species presents a combination of features not found in any other taxon, and attempts to interpret its phylogenetic position within the hominin clade have proved difficult. There are remarkably modern features such as the morphology of the foot^3^, as well as the morphology of the wrist and the relative length of the thumb^4^. However, the cranial capacity is small, both absolutely and relative to body size^1,2^, and there are primitive *Australopithecus*-like traits in the fingers^4^, upper and lower limbs^5,6^ and pelvis^7^. Studies of the dental evidence have likewise revealed a unique combination of features. The permanent postcanine dentition is characterized by small teeth that retain principal molar cusps, but seemingly lack accessory crown traits common in other African hominin groups^1,8^. However, the deciduous dentition shows a number of derived *Paranthropus*-like traits^9^, and molar root metrics find similarities between *H. naledi* and South African *Homo* specimens SK 15 and SK 45, as well as KNM-ER 1805 from Koobi Fora^10^.

Mandibular premolar morphology is particularly useful in studies of hominin taxonomy^11–15^, and initial descriptions suggest the P_3_ of *H. naledi* is highly distinctive. The tooth is absolutely small in size, double rooted, fully bicuspid, and has a symmetrical occlusal outline, a combination of features suggested to be unique among the hominin fossil record^1^. Recent morphometric studies of the mandibular premolar enamel-dentine junction (EDJ) demonstrate that this method has the potential to be a powerful tool in distinguishing between hominin taxa^16,17^. We therefore aim to further investigate this distinctive premolar morphology at the EDJ.

We quantitatively assess the EDJ morphology of the *H. naledi* mandibular premolars (P_3_ and P_4_) using geometric morphometrics (GM), and compare with specimens of *Australopithecus, Paranthropus* and *Homo*. In addition to the *H. naledi* teeth from the Dinaledi Chamber, our sample includes two worn teeth from the Lesedi chamber of the Rising Star Cave system^2^.

We also include specimens from southern Africa that are key to elucidating the systematic placement of *H. naledi*. For example, Stw 80 consists of a crushed mandible and associated teeth from Sterkfontein Member 5 West. The specimen is suggested to resemble SK 15^18^ and is assigned to *Homo* but not given a specific designation. SKX 21204, a juvenile mandibular fragment from Swartkrans Member 1 (Lower Bank), shows a number of features that distinguish the specimen from *Paranthropus*^19^. However, as with Stw 80, it has been assigned to *Homo* but not given a species level designation. Stw 151 (Sterkfontein Member 4) preserves the skull and dentition of a juvenile that shows an overall affinity to *A. africanus*, but shows several derived early *Homo* features, particularly in cranial morphology^20^. SK 96 is a mandible fragment traditionally assigned to *Paranthropus*, but whose P_3_ and canine differ in some respects from other *P. robustus* specimens^21–23^. Finally, the Cave of Hearths mandible, while poorly dated, is attributed to *Homo* and probably antedates the Dinaledi specimens of *H. naledi* by several hundred thousand years^24,25^. It has not been assigned to a species, and Berger and colleagues have suggested the need for comparisons with *H. naledi*^26^. Thus, the diversity of *Homo* species, and their phylogenetic relationship to both *Australopithecus* and *Paranthropus* in southern Africa remains a topic of debate and in this study we examine for the first time the taxonomic signal in mandibular premolar morphology in these specimens and *Homo naledi*.

## Materials and methods

### Study sample

The study sample is summarised in Table 1 (full details can be found in Supplementary Table 1) and consists of 97 teeth (52 P_3_s and 45 P_4_s) from a range of hominin taxa, including 11 *H. naledi* premolars. The sample was chosen to cover taxa endemic to southern Africa (*P. robustus* and *Australopithecus africanus*), groups that have been suggested based on other aspects of the morphology to share a close resemblance with *H. naledi* (early *Homo*), as well as later *Homo* (*Homo neanderthalensis* and *Homo sapiens*) to allow us to identify traits that are primitive and derived for the genus. The early *Homo* sample used here includes specimens from eastern Africa that have been assigned to either *H. erectus* (KNM-ER 992 and KNM-ER 1507) or *H. habilis* (KNM-ER 1802, OH7, OH13), as well as SKX 21204 from Swartkrans and Stw 80 from Sterkfontein that have been assigned to *Homo* but not given a specific designation. Moreover, three specimens whose taxonomic position is uncertain were here designated as indeterminate. These are Stw 151, Cave of Hearths and SK 96.

**Table 1:** Sample summary.

Taxa	Locality	P_3_/P_4_	Complete^a^	Worn^a^
*A. africanus*	Sterkfontein, South Africa	P_3_	5	5
		P_4_	8	8
*P. robustus*	Drimolen and Swartkrans, South Africa	P_3_	5	9
		P_4_	8	8
Early *Homo*	Koobi Fora, Kenya; Swartkrans and Sterkfontein, South Africa; Olduvai Gorge, Tanzania	P_3_	7	7
		P_4_	5	6
*H. naledi*	Rising Star, South Africa	P_3_	4	7
		P_4_	3	4
*H. neanderthalensis*	Combe Grenal and Le Regourdou France; Krapina, Croatia; Scladina, Belgium	P_3_	10	10
		P_4_	8	8
Recent *H. sapiens*	Anatomical collection, various locations	P_3_	11	11
		P_4_	10	10
Indeterminate^b^	Sterkfontein, Cave of Hearths, and Swartkrans, South Africa	P_3_	3	3
		P_4_	1	1

The hominin taxa included in the sample are listed, along with the locality in which they were found, and the number of P_3_s and P_4_s that were included in each of the geometric morphometric analyses.

^a^The complete analysis utilises all landmarks, while the worn analysis uses only a subset corresponding to those preserved in the premolars from the Lesedi (LES) chamber—see main text for full details). A full specimen list can be found in Supplementary Table 1.

^b^Indeterminate specimens are Stw 151 (P_3_ and P_4_), Cave of Hearths (P_3_), and SK 96 (P_3_).

SK 96 consists of a small mandible fragment with the roots of the first deciduous molar, as well as a permanent canine and third premolar. The specimen is often assigned to *Paranthropus robustus* but has been the focus of taxonomic debate. The premolar is unerupted, and Tobias^23^ suggested that incomplete enamel deposition, along with a crack in the crown, meant that a reported high shape index for this tooth (within the range of *Homo habilis*) was not accurate. The associated canine was suggested to be particularly modern in morphology, with Robinson^21^ suggesting that the tooth would have been classified as *Telanthropus* were it not for the morphology of the premolar. Microtomography allows us to digitally remove the crack through the premolar crown and to establish that, rather than being an incomplete germ, the enamel cap is fully formed with the exception of a very small portion of the cervix. The specimen was reconstructed to account for these factors, with several alternate reconstructions tested, details of which can be found in Supplementary Note 1 and Supplementary Figure 1.

### Terminology

The terminology used here to describe premolar morphology follows that of Davies and colleagues^16^ and is outlined in Supplementary Figure 2. Terms refer to the EDJ rather than the OES unless otherwise specified. Crown height at the EDJ can be divided into two components, referred to here as dentine body height and dentine horn height. Dentine body height refers to the distance between the cervix and the marginal ridge(s) that encircle the occlusal basin, while dentine horn height refers to the distance between the marginal ridges and the tip of the tallest dentine horn. Total crown height is the combination of the two.

### Microtomography

Microtomographic scans of the premolar sample were obtained using either a SkyScan 1,173 at 100–130 kV and 90–130 microA, a BIR ACTIS 225/300 scanner at 130 kV and 100–120 microA, a Diondo d3 at 100–140 kv and 100–140 microA and reconstructed as 16-bit tiff stacks, or Nikon XTek at 75 kV and 110 microA (isometric voxel resolutions ranging from 13–45 microns).

### Image filtering

The image stacks for each premolar were filtered in order to facilitate the segmentation of enamel from dentine. Two filters were applied; a three-dimensional median filter and a mean of least variance filter, both with a kernel size of one or three. The kernel size was decided manually by assessing the level of contrast between enamel and dentine in the original scan (those with lower contrast required a kernel size of three). This process improves the homogeneity of the greyscale values for the enamel and dentine, and sharpens the boundaries at the interface between tissue types^27^, and the effect of this process on the morphology of the EDJ has previously been shown to be minimal^28^. Filters were implemented using MIA open source software^29^.

### Tissue segmentation and landmark collection

The filtered image stacks were processed using Avizo 6.3 (https://www.vsg3D.com) in order to produce surface models of the EDJ. Enamel and dentine were segmented using a semi-automatic process that separates voxels based on greyscale values. In some cases, tissue classes are less distinct even after image filtering, making segmentation difficult or impossible using this method. In these cases, a seed growing watershed algorithm was employed via a custom Avizo plugin to segment enamel from dentine, before being checked manually. Once enamel and dentine have been segmented, a triangle based surface model of the EDJ was produced using the unconstrained smoothing parameter in Avizo, and saved in polygon file format (.ply).

In some cases, dental wear removed dentine horn tips. Where this wear was minimal and multiple observers were confident of the dentine horns original position, dentine horns were reconstructed using surface modification tools in Geomagic Studio 2014 (https://www.geomagic.com). This was restricted to cases in which the wear was less than wear level 3 as defined by Molnar^30^.

### Landmark collection and derivation of homologous landmark sets

3D landmarks were collected in Avizo 6.3, and homologous landmarks were derived using a software routine written by Philipp Gunz^31,32^ implemented in Mathematica 10.0 (https://www.wolfram.com). This was done following a previously described protocol^16^ outlined in Supplementary Figure 2.

### Inclusion of specimens from Lesedi Chamber

Two *H. naledi* premolars from the Lesedi Chamber (a third and fourth premolar from the LES1 mandible) show substantial wear such that the dentine horns are almost entirely missing. Therefore, a separate analysis was run in which landmarks corresponding to the worn regions of these teeth were not included, and only the shape of the remaining portion of the EDJ ridge, as well as the cementum-enamel junction (CEJ) ridge, were included. This was done separately for P_3_s and P_4_s to reflect the slightly different patterns of wear in the two teeth. A further two worn P_3_s from the Dinaledi chamber were also included in this analysis to increase the sample size, however no further P_4_s were included.

### Analysis of EDJ and CEJ shape and size

A principal components analysis (PCA) was carried out using the Procrustes coordinates of each specimen in shape space. This was completed separately for P_3_s and P_4_s, firstly utilising all landmarks (*complete* analysis), then subsequently with only the landmarks preserved on the Lesedi specimens (*worn* analysis). The specimens included in each analysis are listed in Supplementary Table 1.

The size of specimens was analysed using the natural logarithm of centroid size and visualised using boxplots. We also tested for differences between *H. naledi* and the other taxa using permutation tests for shape (using Procrustes coordinates) and separately for size (using the natural logarithm of centroid size). Permutation tests were carried out in Mathematica 10.0, using 100,000 permutations. A pooled *Homo* sp. sample was used in order to maximise sample size, and the Benjamini–Hochberg procedure was used to control the false discovery rate^33^. The permutation test, as well as the centroid size boxplot, used data from the *worn* analysis in order to maximise the sample size of *H. naledi*.

### Visualisation of EDJ shape variation

PCA plots of the first two principal components (PCs) were generated separately for P_3_s and P_4_s for both the *worn* and *complete* analyses, for the purpose of displaying the variation present in the sample. Further, surface warps were used to visualise the shape changes along the first two PCs in the *complete* analysis for P_3_s and P_4_s. Here, a reference EDJ surface was created for both the P_3_ and P_4_, which was warped to display the morphology of a hypothetical specimen occupying the extreme ends of each PC, defined as two standard deviations from the mean. The surface warps were generated using Mathematica 10.0 and imaged in Avizo 6.3.

## Results

### Complete analysis

**Figure 1:**
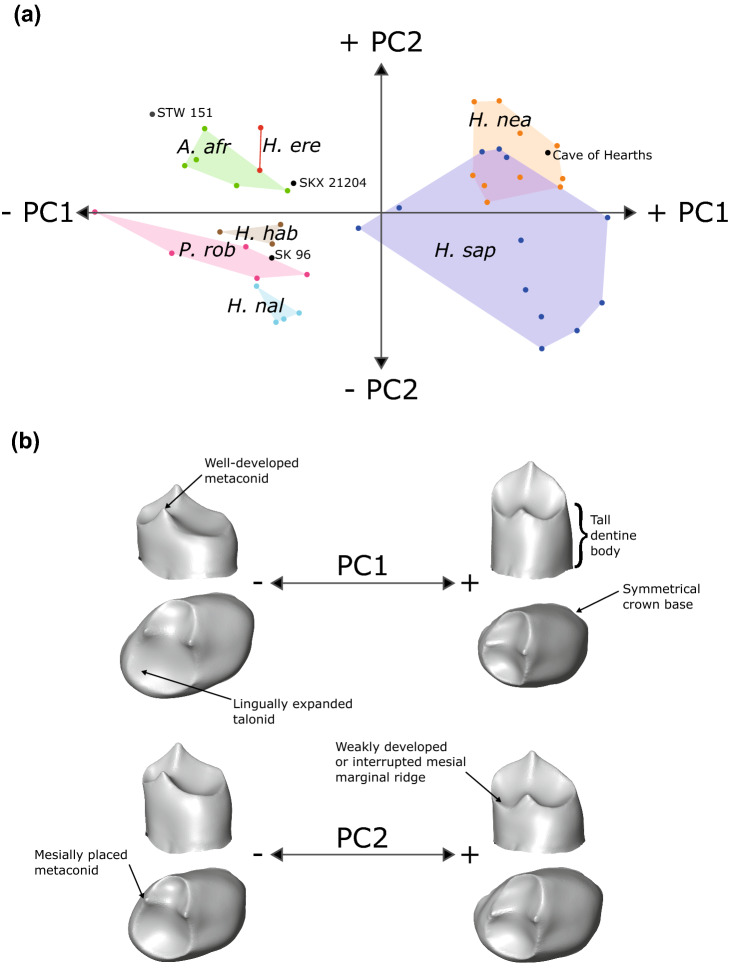
P_3_ EDJ shape variation—*complete* analysis. (**a**) PCA plot showing the first two principal components (PCs) of variation in P_3_ EDJ and CEJ shape. PC1 = 61.2% total variation, PC2 = 12.5%. (**b**) Surface warps depicting the morphological changes captured by each principal component, with labels indicating a number of important features. *A. afr* = *Australopithecus africanus*; *H. ere* = *Homo erectus*; *H. hab* = *Homo habilis*; *H. nal* = *Homo naledi*; *H. nea* = *Homo neanderthalensis*; *H. sap* = *Homo sapiens*; *P. rob* = *Paranthropus robustus*. Surface warp images were created in Avizo 6.3 (https://www.vsg3D.com).

**Figure 2:**
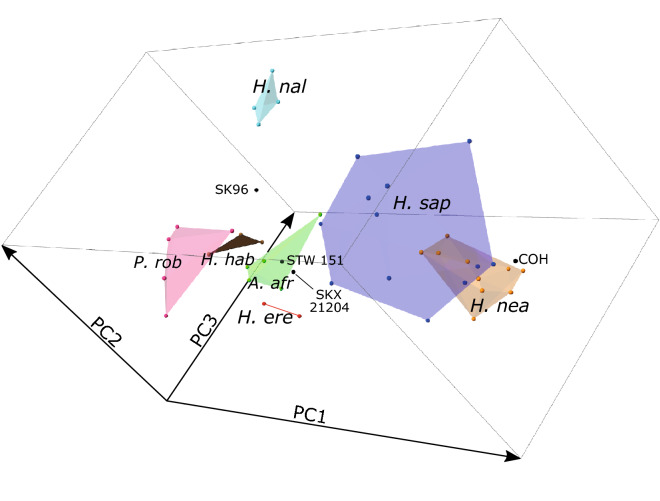
P_3_ EDJ shape variation—*complete* analysis. PCA plot showing the variation in the first three principal components, highlighting the position of SK 96 along PC3. PC1 = 61.2% total variation, PC2 = 12.5%, PC3 = 6.4%. *A. afr* = *Australopithecus africanus*; *H. ere* = *Homo erectus*; *H. hab* = *Homo habilis*; *H. nal* = *Homo naledi*; *H. nea* = *Homo neanderthalensis*; *H. sap* = *Homo sapiens*; *P. rob* = *Paranthropus robustus.*

Principal component analysis of EDJ and CEJ shape reveals that *H. naledi* P_3_s are distinct from other hominin taxa (Fig. 1a). PC1 separates modern humans, Neanderthals and the Cave of Hearths individual from earlier taxa and *H. naledi*. This is largely driven by the taller dentine body height seen in later taxa, the reduction of the talonid region, a reduced metaconid, and a symmetrical crown base (Fig. 1b). For this principal component, all *H. naledi* specimens occupy a similar range to early *Homo* specimens, as well as some *P. robustus* and *A. africanus* specimens. This placement reflects the presence in *H. naledi* P_3_s of a moderately tall dentine body height, a talonid that is somewhat expanded, and an asymmetrical crown base. *Homo naledi* occupies the negative end of PC2, which separates the species from other fossil hominin taxa. This separation is driven largely by a combination of a high mesial marginal ridge and a mesially placed metaconid, relative to other taxa. PC2 is particularly important in separating *H. naledi* from *H. erectus* and Swartkrans *Homo* specimen SKX 21204, as well as, to a lesser extent, *H. habilis*. Stw 151 is well separated not just from *H. naledi*, but from all early *Homo* specimens included here, instead falling closest to *A. africanus*. *Paranthropus robustus* falls closest to *H. naledi*, reflecting the shared presence of a number of the aforementioned features, including an asymmetrical crown base, a mesially placed metaconid and a high mesial marginal ridge. In the first two principal components, some *P. robustus* specimens plot particularly close to *H. naledi*, however all are well separated in PC3 (Fig. 2). Within the space of the first three PCs, SK 96 falls intermediate between *P. robustus* and *H. naledi*. This intermediate position reflects the presence in SK 96 of a combination of features; aligning the specimen with *H. naledi* are the somewhat reduced talonid, mesiodistally expanded occlusal basin and buccolingually narrower crown base, relative to *P. robustus*. However, the specimen also has a smaller metaconid than is typical of *H. naledi*, which is seen in some *P. robustus* specimens.

**Figure 3:**
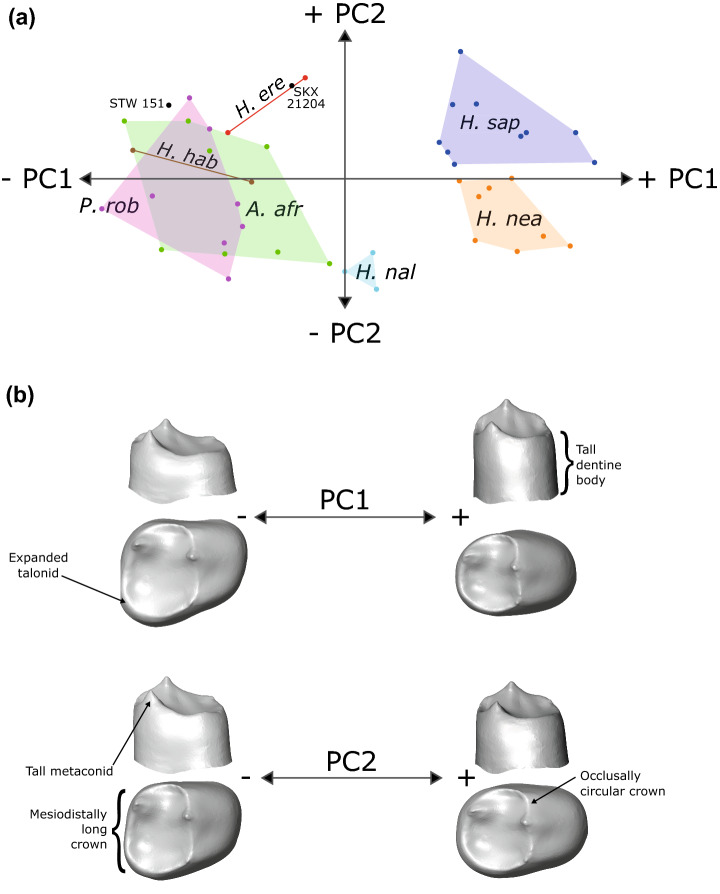
P_4_ EDJ shape variation—*complete* analysis. (**a**) PCA plot showing the first two principal components (PCs) of variation in P_4_ EDJ and CEJ shape. PC1 = 56.7% total variation, PC2 = 10.3%. (**b**) Surface warps depicting the morphological changes captured by each principal component, with labels indicating a number of important features. *A. afr* = *Australopithecus africanus*; *H. ere* = *Homo erectus*; *H. hab* = *Homo habilis*; *H. nal* = *Homo naledi*; *H. nea* = *Homo neanderthalensis*; *H. sap* = *Homo sapiens*; *P. rob* = *Paranthropus robustus*. Surface warp images were created in Avizo 6.3 (https://www.vsg3D.com).

When looking at P_4_s, PC1 again separates modern humans and Neanderthals from other taxa (Fig. 3a), while PC2 distinguishes between modern humans and Neanderthals. *Homo naledi* occupies an intermediate position along PC1, which, as in the P_3_, is largely driven by dentine body height and talonid development (Fig. 3b). Although the talonid in *H. naledi* is relatively large, as in *P. robustus*, *A. africanus* and early *Homo*, the dentine body is taller than in these groups, which explains its intermediate position along the PC1. While PC2 does not distinguish between *P. robustus* and *A. africanus*, it does separate *H. naledi* from early *Homo* specimens, particularly *H. erectus* and SKX 21204. This is influenced by the height of the metaconid, as well as the length of the occlusal basin in the mesiodistal direction. In the case of *H. naledi*, the metaconid is tall, as in the P_3_, and the crown is mesiodistally expanded. This is particularly noticeable when comparing with *H. erectus* from Koobi Fora and SKX 21204, which have mesiodistally short crowns that are roughly circular in occlusal view. Another feature that contributes to PC2 and distinguishes between *H. naledi* and early *Homo* specimens is the buccolingually narrow shape of the *H. naledi* crown base.

### Worn analysis

**Figure 4:**
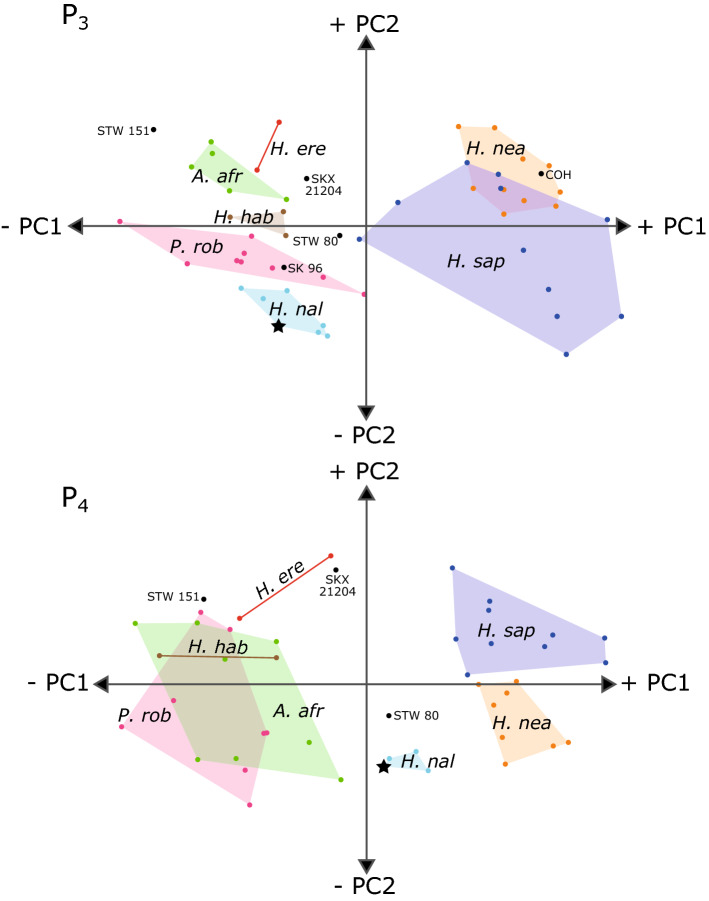
PCA plots of EDJ shape variation – *worn* analysis including a P_3_ and P_4_ from the Lesedi Chamber (marked with stars). Lesedi specimens are particularly worn, so only landmarks representing preserved regions in these specimens were included here. P_3_ PC1 = 57.3% total variation, PC2 = 11.5%; P_4_ PC1 = 56.5%, PC2 = 12.1%. *A. afr* = *Australopithecus africanus*; *H. ere* = *Homo erectus*; *H. hab* = *Homo habilis*; *H. nal* = *Homo naledi*; *H. nea* = *Homo neanderthalensis*; *H. sap* = *Homo sapiens*; *P. rob* = *Paranthropus robustus.*

As the LES1 premolars are worn beyond the stage where the dentine horns could be reconstructed, landmarks that correspond to worn regions of the crown were dropped from the analysis. Importantly, landmarks and semilandmarks for the protoconid and metaconid are excluded, meaning that the height of the dentine horns is not considered but dentine body height is captured. PCA plots for this analysis are shown in Fig. 4, where it can be seen that both LES1 premolars (P_3_ and P_4_) cluster closely with those of the Dinaledi chamber, indicating that there is little difference in premolar morphology between individuals from the two chambers. Further, the overall distribution of hominin specimens remains largely the same as in the *complete* analysis, albeit with minor differences, for both P_3_s and P_4_s (Figs. 1 and 3). *Homo naledi* specimens are still distinct from other hominin taxa, and the Cave of Hearths specimen of uncertain affinity, in the *worn* analysis, suggesting that the height of the protoconid and metaconid are not the only aspects of premolar shape that contribute to the observed patterns. This analysis also allows the inclusion of the worn Stw 80 premolars. The Stw 80 P_3_ falls closest to modern humans in Fig. 4, however it is also relatively close to the *H. habilis* range of variation. It is well-separated from Koobi Fora *H. erectus* specimens and Stw 151, although it is closer to SKX 21204. The Stw 80 P_4_ falls closer to the *H. naledi* range of variation and is well-separated from *H. erectus* and *H. habilis* specimens, as well as SKX 21204, Stw 151, and Cave of Hearths.

The permutation tests for P_3_ and P_4_ shape were completed using the *worn* analysis in order to increase sample sizes, particularly for *H. naledi*. This showed that the *H. naledi* P_3_ can be statistically distinguished from all other taxa in shape, including the combined early *Homo* sample (Table 2). For the P_4_ shape, *H. naledi* was found to differ from all other taxa except *H. neanderthalensis* and the pooled early *Homo* sample. Neanderthals are clearly distinct from *H. naledi* in Fig. 4, and only STW 80 is close to the *H. naledi* range of variation in the early *Homo* sample, so it is possible that a larger sample size of *H. naledi* P_4_s may have allowed these groups to be distinguished statistically.

**Table 2:** Permutation tests for shape (using Procrustes coordinates) and centroid size to test for differences between *H. naledi* and the four comparative taxa.

*H. naledi vs…*	*A. africanus*	*P. robustus*	*Homo* sp.	*H. neanderthalensis*	*H. sapiens*
P_3_ shape	**0.018**	**0.045**	**0.030**	**0.015**	**0.016**
P_3_ size	**0.031**	**0.001**	0.072	0.395	**0.001**
P_4_ shape	**0.018**	**0.015**	0.060	0.111	**0.025**
P_4_ size	**0.010**	**0.010**	**0.018**	0.395	**0.015**

The Lesedi (subset) landmark data was used here to maximise sample size and to allow inclusion of the LES1 specimens. Bold indicates *p* < 0.05.

### Size

**Figure 5:**
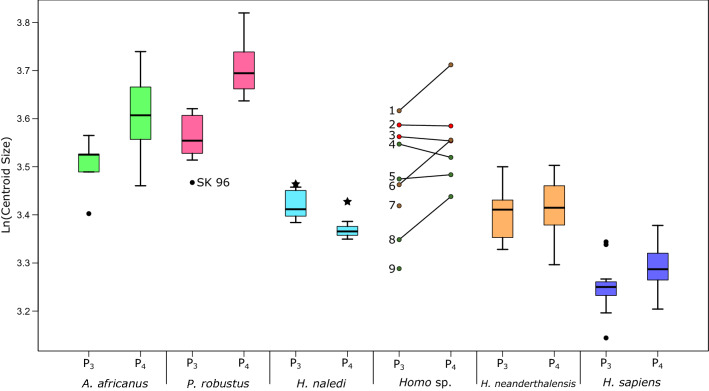
Boxplot of premolar centroid size. Plots show the natural logarithm of centroid size for the P_3_ and P_4_ of each taxon in the landmark drop analysis, as well as four specimens not assigned to any of these taxa. *H. naledi* specimens from the Lesedi Chamber are separated from the Dinaledi chamber sample and are marked with a star. *Homo* sp. specimens are (1) KNM-ER 1802; (2) KNM-ER 992; (3) KNM-ER 1507; (4) STW 80 5) STW 151 (6) OH7; (7) OH13; (8) SKX 21204; (9) Cave of Hearths. Note: STW 151 and Cave of Hearths are considered taxonomically indeterminate in this study, however they are displayed with *Homo* sp. here for comparative purposes.

Specimen size was analysed using the centroid sizes calculated in the *worn* analysis, again to maximize sample sizes. Figure 5 shows a boxplot of these results, and the results of the permutation test for differences between each taxon and *H. naledi* can be found in Table 2. *Homo naledi* specimens are small compared to those of other taxa; the P_3_ and P_4_ are significantly smaller than those of *P. robustus* and *A. africanus,* while the P_4_s are also significantly smaller than the combined *Homo* sample. Both premolars are significantly larger than *H. sapiens,* and are approximately the same size as those of Neanderthals. The LES1 premolars are slightly larger than the Dinaledi specimens included here; this is more noticeable for the P_4_ than the P_3_, although there are fewer Dinaledi P_4_s in the sample and it is possible that the inclusion of more specimens would change this.

Interestingly, *H. naledi* is the only taxon included here for which the mean P_4_ size is markedly smaller than the mean P_3_ size, a pattern present in both Dinaledi and Lesedi premolar pairs Two *H. erectus* pairs from Koobi Fora, KNM-ER 992 and KNM-ER 1507, show a sub-equal size difference, while in *H. habilis* individuals OH7 and KNM-ER 1802 the P_4_ is clearly larger. However, this pattern is also reproduced in Sterkfontein *Homo* specimen STW 80, while STW 151 has approximately the same size P_3_ and P_4_.

## Discussion

The mandibular premolars of *H. naledi*, particularly the P_3_, were described as showing several distinctive features^1^. We find this distinctiveness is also evident at the EDJ, with a distinct cluster of *H. naledi* premolars separated from all other taxa in EDJ shape, and consistently displaying a suite of distinctive features (Supplementary Figure 3). This includes a high P_3_ mesial marginal ridge, a tall mesially placed P_3_ metaconid, and a mesiodistally expanded P_4_ crown. Further, the premolars from the Lesedi Chamber cluster closely with the Dinaledi Chamber specimens in shape space (Fig. 4). Although the centroid size of both LES1 premolars is outside the range of the Dinaledi specimens, the difference is very small for the P_3_. There is a larger difference for the P_4_, however only a small number of Dinaledi P_4_s were included, and it is possible that this difference would not be maintained with a larger sample, particularly as there is no size difference evident from linear measurements of teeth from the two chambers^1, 2^. Previous studies have suggested that *H. naledi* may have occupied a unique dietary niche consisting of foods with a high level of dust/grit contaminants, as reflected in the wear resistance of the molars^34^ and the high level of dental chipping^35^. Although studies of the EDJ cannot address these hypotheses directly, our results would be consistent with *H. naledi* occupying a dietary niche distinct from that seen in other hominin groups.

Previous studies have noted low levels of variation within the *H. naledi* sample when compared with other hominin taxa^1,2,34,36^. We similarly find a homogenous EDJ morphology in *H. naledi* premolars both within the Dinaledi Chamber, and between the Dinaledi and Lesedi chambers. A previous study of the P_3_ EDJ including a broader sample of fossil hominins and extant apes supports the suggestion that *H. naledi* is unusual in its homogeneity, with seemingly less variation in size and shape than a subspecies of chimpanzee (*Pan troglodytes verus*)^16^, which has itself been suggested to be less variable in dental morphology than other *Pan* subspecies^37^. Dental morphology is thought to be highly heritable, so it is possible that this low level of dental variation reflects low genetic diversity. In African apes, it seems that population structure is important in understanding levels of dental variation; gorillas show hierarchical levels of dental variation such that there is more variation at the species than subspecies level, while in chimpanzees the subspecies and species levels of variation are roughly similar. Similarly, in modern humans there appears to be high levels of intra-population variability in premolar EDJ shape^38^. In sum, low levels of dental variability could suggest the *H. naledi* remains are from relatively closely related individuals in a single population. However, we also cannot rule out that there are also functional or ecological factors contributing to this uniformity, or that a larger sample of *H. naledi* premolars would reveal a larger degree of variation than seen in this study. A study of EDJ morphology among extant individuals of known relatedness would be useful in interpreting levels of EDJ variation in fossil taxa.

The *H. naledi* premolars are small, overlapping with those of Neanderthals. The P_4_s, in particular, are small relative to the sample of *Australopithecus*, *Paranthropus*, *H. erectus* from Koobi Fora (KNM-ER 992, KNM-ER 1507), and *H. habilis* (OH 7 and KNM-ER 1802). The size relationship between the premolars in *H. naledi* is unusual amongst the hominin sample used here. *Australopithecus africanus*, *P. robustus* and *H. habilis* have a larger P_4_ than P_3_ (this pattern is evident from the median for each group as shown in Fig. 5, and is consistent among individuals for which both premolars are preserved—Supplementary Table 1), while Neanderthals and modern humans have premolars that largely overlap in size. However, in *H. naledi* the mean P_4_ size is smaller than that of the P_3_.

Previous studies find that some African and European Pleistocene *Homo* groups display a P_3_ > P_4_ ratio when considering planimetric crown area, including *H. ergaster*^39,40^, we do find that the P_4_ is smaller in KNM-ER 992 and KNM-ER 1507, however the difference is extremely small. Using centroid size of the partial EDJ is likely to give somewhat different results to planimetric area or linear dimensions since the thickness of the enamel is not considered, and size of the dentine crown is measured in three dimensions. Interestingly, although STW 80 has overall larger premolars than *H. naledi*, it is the only early *Homo* specimen to show a P_3_ > P_4_ pattern. However, relatively few specimens in the comparative sample preserve a P_3_ and a P_4_, so further investigation is required to determine the consistency of this pattern within individuals.

Variation in P_3_:P_4_ size has been studied by a number of authors^22,40–42^ and may have particular taxonomic importance. Some have suggested that the anterior and posterior dentitions are somewhat independent, and that in this case the P_4_ would covary with the molars, while the P_3_ would covary more with the canine. In this case however, we would expect the *H. naledi* canines to be relatively large, which is not the case^2^. More recently, mouse models have suggested the existence of an inhibitory cascade model in which the size of a tooth is dependent on inhibition from previously developing teeth. The size of the primary postcanine teeth (deciduous molars and permanent molars) can be understood in this context^43^, however the situation is more complex for mandibular premolars as they develop after the deciduous molars, permanent canine and permanent first molar. A study considering the size of the entire tooth row would be necessary to investigate whether or not the observed tooth size patterns in *H. naledi* fit the expectations of this model.

Despite abundant features throughout the *H. naledi* skeleton that are reasonably interpreted as primitive for *Homo*, the premolars of *H. naledi* evince a number of very clear differences from the majority of the early *Homo* specimens included here. Moreover, we found a significant difference between *H. naledi* and a combined early *Homo* group in P_3_ shape (Table 2). For both P_3_ and P_4_, PC2 distinguishes between *H. naledi*, *H. habilis* and *H. erectus*, with *H. erectus* and *H. naledi* occupying the extremes. This axis relates to the placement of the metaconid in both premolars (mesial in *H. naledi*, distal in *H. erectus*), the development of the P_3_ mesial marginal ridge (high in *H. naledi*, lower in *H. erectus*), the relative mesiodistal length of the P_4_ crown (longer in *H. naledi*, shorter in *H. erectus*) and the relative buccolingual width of the crown base in both premolars (narrower in *H. naledi*, longer in *H. erectus*). In this respect, *H. habilis* is intermediate, and much more closely resembles the *Australopithecus* condition (Figs. 1 and 3), as would be expected for a species basal to the genus *Homo*. Our results suggest that the two derived premolar morphologies represented by *H. naledi* and *H. erectus* could have evolved separately from a more generalised *H. habilis*-like ancestral condition. The alternative explanation of character transformation series that resembles *H. habilis–H. erectus–H. naledi*, would require reversals in a number of the features mentioned above. However, *H. naledi* postdates the *H. erectus* specimens included here by around 1.5 million years, which would be more than enough time for these changes to take place. Further, the sample used here is limited with respect to early *Homo*, so we should be cautious in these conclusions.

Swartkrans specimen SKX 21204 has been assigned to *Homo*^19^, but has not been given a specific designation. Here the P_3_ and P_4_ cluster more closely to eastern African *H. erectus* than *H. habilis*, with the P_4_ particularly close to KNM-ER 992 in shape space (Fig. 3). Although SKX 21204 is small, similar to *H. naledi* when considering centroid size (Fig. 5), size appears to be variable in a number of taxa, and the shape of the premolars suggests that (1) the specimen is clearly distinguished from *H. naledi* and (2) among our sample, the specimen is a good match for African *H. erectus*. STW 151 was suggested to possibly represent an individual more derived towards *Homo* than other Sterkfontein *A. africanus* specimens^20^. Here we find that both P_3_ and P_4_ are close to, but not within, the *A. africanus* range of shape variation, and neither premolar shows any particular affinity to early *Homo* specimens, or those of *H. naledi*. However, it should be noted that the features aligning the specimen with early *Homo* were mostly in other areas of the dentition and the cranium, rather than the mandibular premolars. The Cave of Hearths P_3_ most closely resembles Neanderthals, and is clearly distinct from *H. naledi* (for more details on this specimen, see refs 15,16). As with the *H. erectus* specimens, positing the Cave of Hearths specimen as an ancestor of *H. naledi* would entail a number character reversals. The Cave of Hearths P_3_ is better fit as a human ancestral form than it is an ancestral form for *H. naledi*.

Stw 80 is a crushed mandible from Sterkfontein Member 5 West assigned to early *Homo*^44^, and has been suggested to show strong similarities to SK 15 from Swartkrans^18^. We find that the P_3_ morphology is unusual; it has a very large talonid, which is primitive for *Homo*, however it also has a relatively tall dentine body, buccolingually narrow anterior fovea and a large accessory crest in the posterior fovea. This combination of traits is not seen in any other specimens in our sample, and the *worn* analysis does not show clear affinities between the P_3_ and any of the hominin taxa included here. Unfortunately, the protoconid of both P_3_ and P_4_ are worn, precluding assessment of the relative cusp heights, which can be useful in distinguishing between taxa. The P_4_ falls relatively close to *H. naledi* in Fig. 4, driven partly by the combination of a tall dentine body and a mesiodistally expanded crown. This combination distinguishes *H. naledi* from the *H. habilis* and *H. erectus* specimens in our sample, possibly representing a *H. naledi* apomorphy. Equally, Stw 80 shows a P_3_ > P_4_ pattern, which, as discussed earlier, may be a *H. naledi* apomorphy. The shared presence of these derived traits could suggest a phylogenetic relationship between this specimen and *H. naledi*, however further investigation involving the entire tooth row would be necessary to investigate this further as it is possible that the similarities we have outlined are due to homoplasy. It is important to note that while the majority of the teeth of Stw 80 are too damaged to measure, the mesiodistal length of both the canine and M_3_ are larger than in *H. naledi*^1,44^.

The taxon that falls closest to *H. naledi* in P_3_ shape in the first two PCs is *P. robustus* (Fig. 1), which is driven by both taxa sharing a tall mesially placed metaconid and well-developed marginal ridges. The talonid is also somewhat expanded in both taxa, although more so in *P. robustus*. The taxa are separated in PC3 however (Fig. 2), which reflects in part the difference in talonid size, as well as the crown being mesiodistally longer in *H. naledi*. Further, there are size differences between the two taxa (Fig. 5) and the permutation test found them significantly different in both shape and size (Table 2). Equally, the P_4_ of *H. naledi* falls closer to *A. africanus* than *P. robustus* (Fig. 2) and is significantly smaller than both *A. africanus* and *P. robustus* (Table 2, Fig. 5). It is therefore possible that the similarities between these two taxa in P_3_ morphology are due to homoplasy.

**Figure 6:**
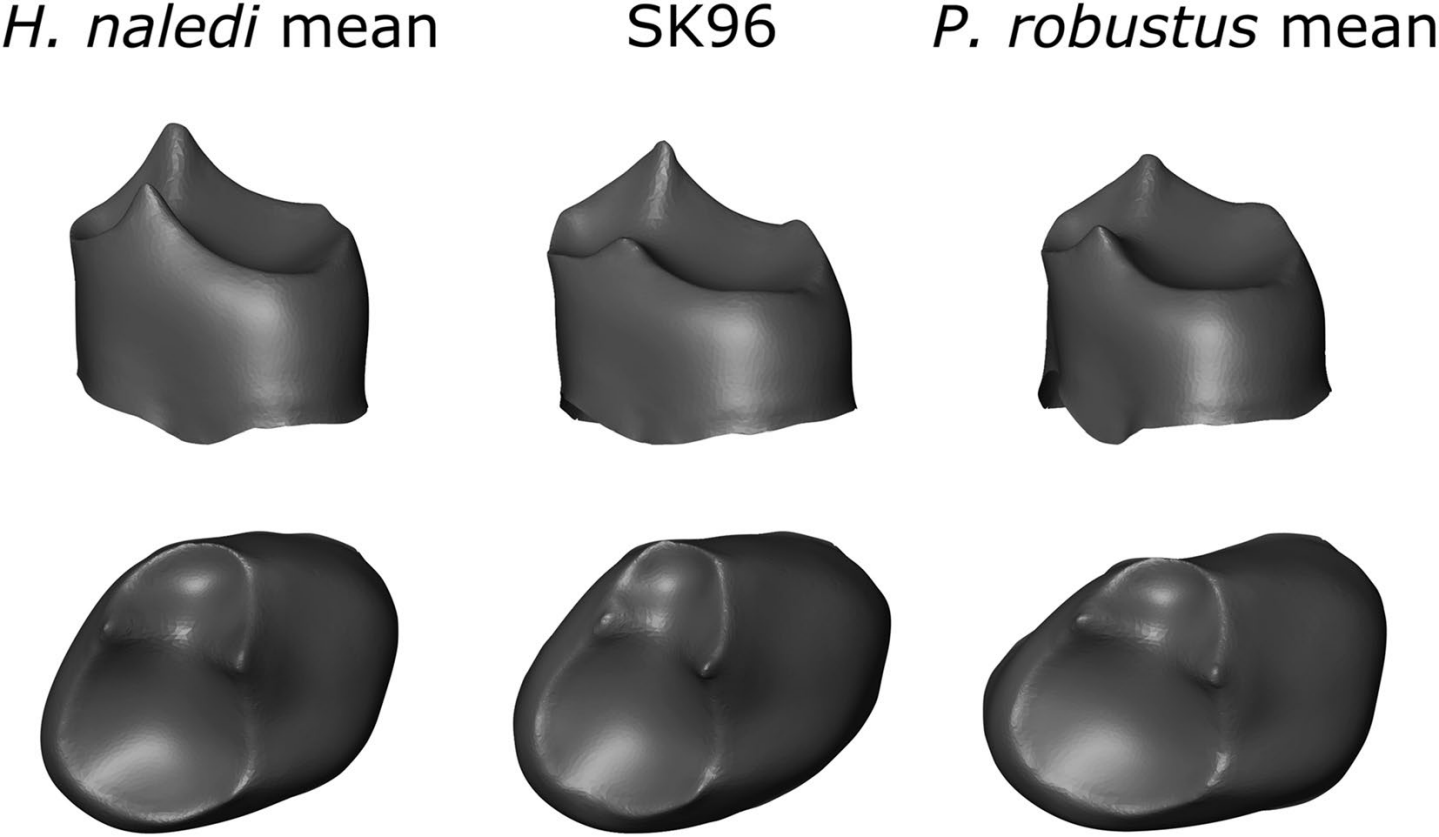
P_3_ surface warps for mean models of *Homo naledi* and *Paranthropus* as well as SK 96. Note, SK 96 is left sided, but the surface warp is here shown as right sided for comparative purposes. Surface warp images were created in Avizo 6.3 (https://www.vsg3D.com).

The picture is more complicated when considering SK 96 however (Fig. 6); the specimen is from Member 1 at Swartkrans, and consists of a mandible fragment, canine and P_3_. The premolar was found to have a more mesiodistally expanded crown than other *P. robustus* P_3_s^21^, however this was attributed to the tooth being incomplete and cracked^23^. After verifying that the crown is all-but complete, and fixing the crack, we find that the specimen is smaller in centroid size than any other *P. robustus* P_3_ included (n = 9), instead falling within the range of the P_3_s of Neanderthals, *H. habilis* and slightly above the *H. naledi* size range. Further, the shape of the EDJ is outside the range of *P. robustus*, instead occupying a space between *P. robustus*, *H. habilis*, and *H. naledi* in the first 3 PCs (Fig. 2). A feature of *Paranthropus* is the very large P_3_ talonid; SK 96 instead has a moderate talonid more similar to *H. habilis* and *H. naledi*. Equally however, SK 96 does not show the clearly well-developed metaconid typical of *H. naledi*, instead showing a smaller metaconid similar to that found in in some *P. robustus* and *H. habilis* specimens.

SK 96 also preserves a lower canine and part of the roots of a deciduous first molar. The canine was described as showing a particularly modern morphology^22^, and is the smallest *P. robustus* specimen in crown dimensions^22,45–47^. The crown size is also smaller than *H. habilis* but is within the range of *H. naledi*. However it does not have the typical *H. naledi* canine morphology (Supplementary Figure 4); the SK 96 crown is less asymmetrical, more rounded, and lacks a distal accessory cuspule^1,2^. The canine of OH 7 and OH 13 are also markedly asymmetrical, and have moderately developed distal cuspules^41^, differentiating them from SK 96.

If SK 96 does belong to *P. robustus*, it would be extreme for the species in premolar shape (Fig. 2) and size (Fig. 5, also see refs^22,45^), as well as canine size^22,45,46^. It would also suggest that traits considered to be characteristic of the species, such as expansion of the P_3_ talonid, are less pronounced in some individuals. Therefore, it is possible that this specimen instead represents *Homo*; although there are differences in canine morphology, the SK 96 P_3_ shares some features with *H. habilis*. Member 1 at Swartkrans contains a number of specimens assigned to *Homo*, including SK 27 and SK 45, and it is possible that SK 96 represents the same taxon as these specimens, as well as SK 15 from Member 2.

Also worthy of note are the similarities between these specimens and *H. naledi* (See Supplementary Figure 5a). SK 96 resembles *H. naledi* in P_3_ morphology more closely than any other specimen included here, while Stw 80 falls closest to *H. naledi* in P_4_ shape, and shares the species’ P_3_ > P_4_ pattern. This would also be consistent with the finding that two key Swartkrans *Homo* specimens (SK 15 and 45) share a number of molar root characteristics with *H. naledi*^10^. On the other hand, SKX 21204 also derives from Swartkrans Member 1 (Lower Bank) and is here found to show a number of clear differences in P_3_ morphology from SK 96 (see Supplementary Figure 5a,b). SKX 21204 is a better fit for *H. erectus* based on premolar morphology, which could suggest the presence of multiple non-*P. robustus* taxa in Swartkrans Member 1. Further investigation is needed to fully assess whether these differences are sufficient to warrant species-level separation.

The similarities between SK 96 and *H. naledi* could be further evidence^10^ for some phylogenetic link between hominins at Swartkrans and *H. naledi*, while the similarities with Stw 80 may suggest a similar link with Sterkfontein hominins. This is striking given that both Swartkrans Member 1 and Sterkfontein Member 5 West are suggested to predate *H. naledi* by more than a million years^18,24,48–50^, and would suggest that *H. naledi* represents a long surviving lineage that split from other members of the genus *Homo* relatively early. In this regard, the Cave of Hearths specimen is notable because it evinces a more human-like morphology and likely antedates the dated *H. naledi* material by hundreds of thousands of years. However, it is also possible that the similarities between these specimens and *H. naledi* are due to homoplasy. It should be noted that although SK 96 and STW 80 show similarities to *H. naledi* individually, their P_3_ morphologies are not especially similar to one another. Overall, the Sterkfontein and Swartkrans early *Homo* assemblage does not appear to be homogenous in premolar morphology. It is therefore important that the remaining dentition and mandibular morphologies of these specimens are also investigated and compared to *H. naledi* where possible to allow us to assess all available evidence.

## Conclusions

Overall, we find that there are a number of aspects of mandibular premolar EDJ morphology that are distinctive in *H. naledi* when compared to a broad sample of hominins, including a number of key early *Homo* specimens. The morphology of the *H. naledi* premolars is highly consistent and homogeneous when compared with other samples included here, and distinctive traits are displayed consistently throughout the collection including a tall well-developed metaconid in both the P_3_ and P_4_, a relatively mesiodistally long P_4_ crown, and strongly developed mesial marginal ridges. The worn LES1 premolars are also consistent with this morphology. This distinctive morphology may be useful in the future in identifying further specimens of *H. naledi*, potentially from limited and fragmentary remains. We also suggest that SK 96, previously attributed to *P. robustus*, differs from the hypodigm in P_3_ EDJ morphology and may instead represent *Homo*. The specific designation of the specimen, and the relationship between this and other South African *Homo* specimens, including Stw 80 from Sterkfontein, requires further investigation.

## Supplementary information

Supplementary Information 1.

## Data Availability

The datasets generated during the current study are available from the corresponding author on reasonable request.
