# Supplementary material for: Distinct mandibular premolar crown morphology in Homo naledi and its implications for the evolution of Homo species in southern Africa
Source: Sci Rep. 2020 Aug 6;10:13196. doi: 10.1038/s41598-020-69993-x (PMC7413389; doi:10.1038/s41598-020-69993-x)
Supplement: Supplementary file 1 — Supplementary Information 1. [file 41598_2020_69993_MOESM1_ESM.pdf]

Supplementary information

Distinct mandibular premolar crown morphology in *Homo naledi* and its implications for the evolution of *Homo* species in southern Africa.

Thomas W. Davies[a,b,*], Lucas K. Delezene[c,d], Philipp Gunz[a], Jean-Jacques Hublin[a,e], Lee R. Berger[d], Agness Gidna[f], Matthew M. Skinner[a,b,d]

[a]*Department of Human Evolution, Max Planck Institute for Evolutionary Anthropology, Deutscher Platz 6, 04103, Leipzig, Germany*

[b]*School of Anthropology and Conservation, University of Kent, Canterbury, CT2 7NZ, United Kingdom*

[c]*Department of Anthropology, University of Arkansas, Fayetteville, Arkansas, 72701 USA*

[d]*Evolutionary Studies Institute, University of the Witwatersrand, 1 Jan Smuts Avenue, Braamfontein, 2000, Johannesburg, South Africa*

[e]*Collège de France, 11 Place Marcellin Berthelot, 75005 Paris, France*

[f]*Paleontology Unit, National Museum of Tanzania, Dar es Salaam, Tanzania*

*Corresponding author. E-mail address: thomas_davies@eva.mpg.de

Supplementary Note – SK 96 background and reconstruction

SK 96, from Swartkrans Member 1, consists of a small mandible fragment with the roots of the first deciduous molar, as well as a permanent canine and third premolar. The SK 96 P_3 has a crack running diagonally across the crown, through the protoconid, that artificually lowers the mesiobuccal portion of the crown. The two portions of the enamel-dentine junction were realigned using the manual registration tool in Geomagic. This process was comlpeted twice, alternating which half of the enamel-dentine junction (EDJ) was used as a reference and which was moved to match. These will be labelled as reconstruction A and B.

Secondly, while the majority of the cervix is fully formed on SK 96, a small portion on the lingual side extends more apically than the rest of the cervix, and appears to be incomplete. This portion of the cervix somewhat resembles the condition seen in double/tomes root specimens in which the enamel extends further apically in the radical between the root sections. However, in this case the enamel extends much further apically and inwards (buccally). To account for this, several landmark placements were completed for this portion of the cervix, and are numbered as follows:

1) The extension of enamel was landmarked as it appears in the specimen, assuming the cervix was complete
2) The landmarks positions were estimated based on where the cervix would have likely been when fully formed, based on the shape and thickness of the enamel and dentine in the undeveloped section
3) The landmarks positions were estimated as a ‘most extreme’ estimate, in which the distance from the already deposited enamel was increased to test how our estimation impacts the results
4) The landmarks were placed ignoring the enamel extension, instead running directly between portions of the cervix either side that are fully formed.

This results in 8 reconstructions for the specimen in which each of the four cervix reconstructions are repeated for both crack reconstructions. Geometric morphometric analysis of EDJ and CEJ shape, and only cementum-enamel junction (CEJ) shape, were run in order to assess the affect these reconstructions have on the placement of the specimen, and the results are shown in Supplementary Figure 1. The reconstruction of the crack (labelled A and B) has a small impact but does not substantially change the position the specimen occupies in shape space. The cervix reconstructions have a small impact when CEJ and EDJ landmarks are included, but the difference between reconstructions is more substantial when only the CEJ is considered. In this case, reconstructions 1-3, in which the apical extension of the enamel are included, fall apart from all other specimens in the analysis. Reconstruction 4 (A and B), in which the apical extension of enamel is ignored, fall much closer to the other specimens in the sample, suggesting that these are a more realistic placement for the cervical landmarks.

Overall, we suggest that B4 is the best reconstruction. This is because crack reconstruction B involves using the larger EDJ portion as a reference and moving the smaller portion, meaning that fewer landmarks are placed according to the moved portion. Cervix reconstruction 4 was chosen because it results in a cervix landmark configuration that clusters far more closely to other hominin specimens (Supplementary Figure 1).

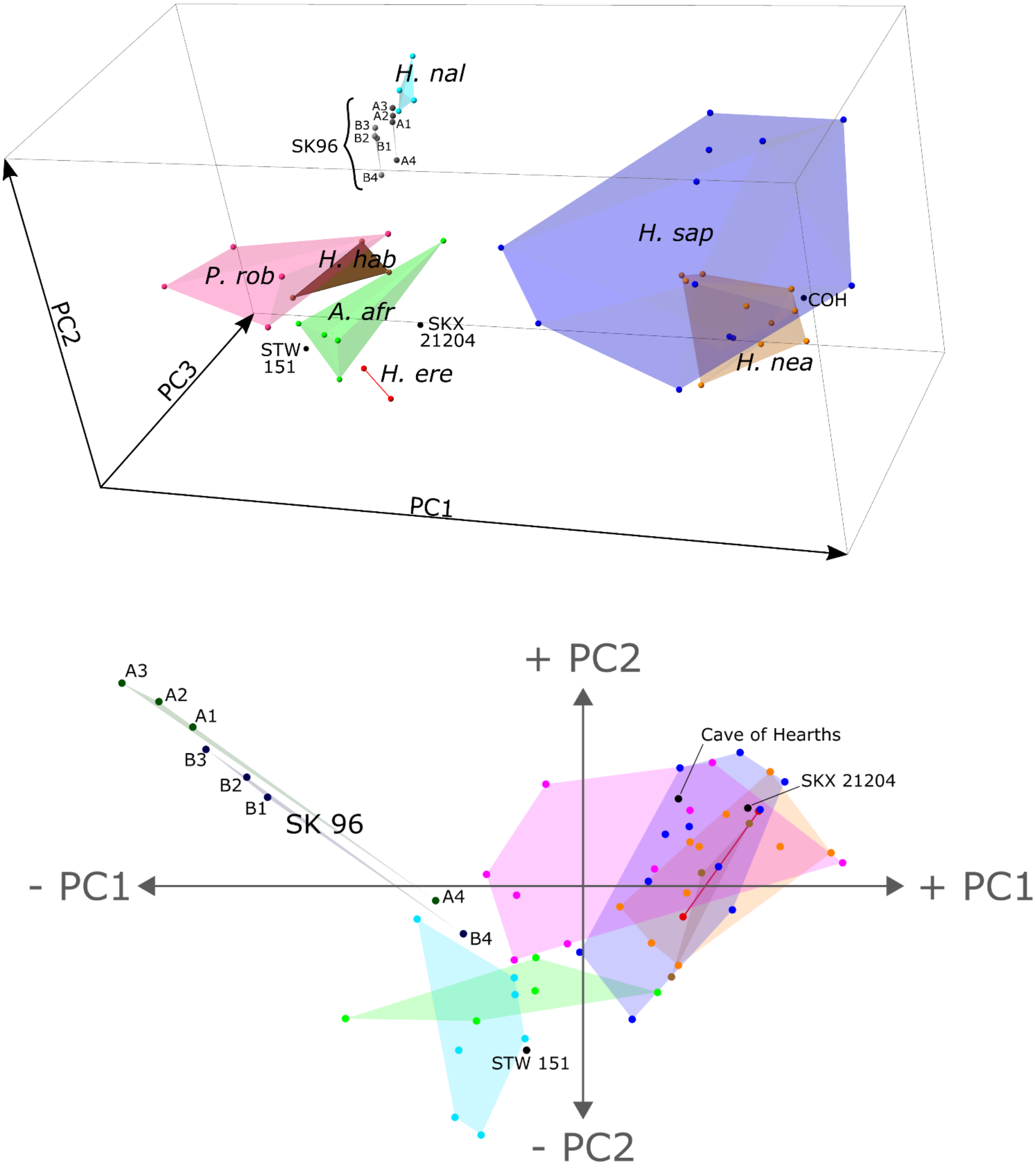

Supplementary Figure 1. PCA plots showing the variation in reconstructions of SK 96. Two crack reconstructions are labelled A and B, and four reconstructions of the cervix are numbered 1-4 (see Supplementary Note 1). Top: 3D PCA of the *complete* landmark set (EDJ and CEJ) with the reconstructions included. Bottom: PCA of CEJ shape only.

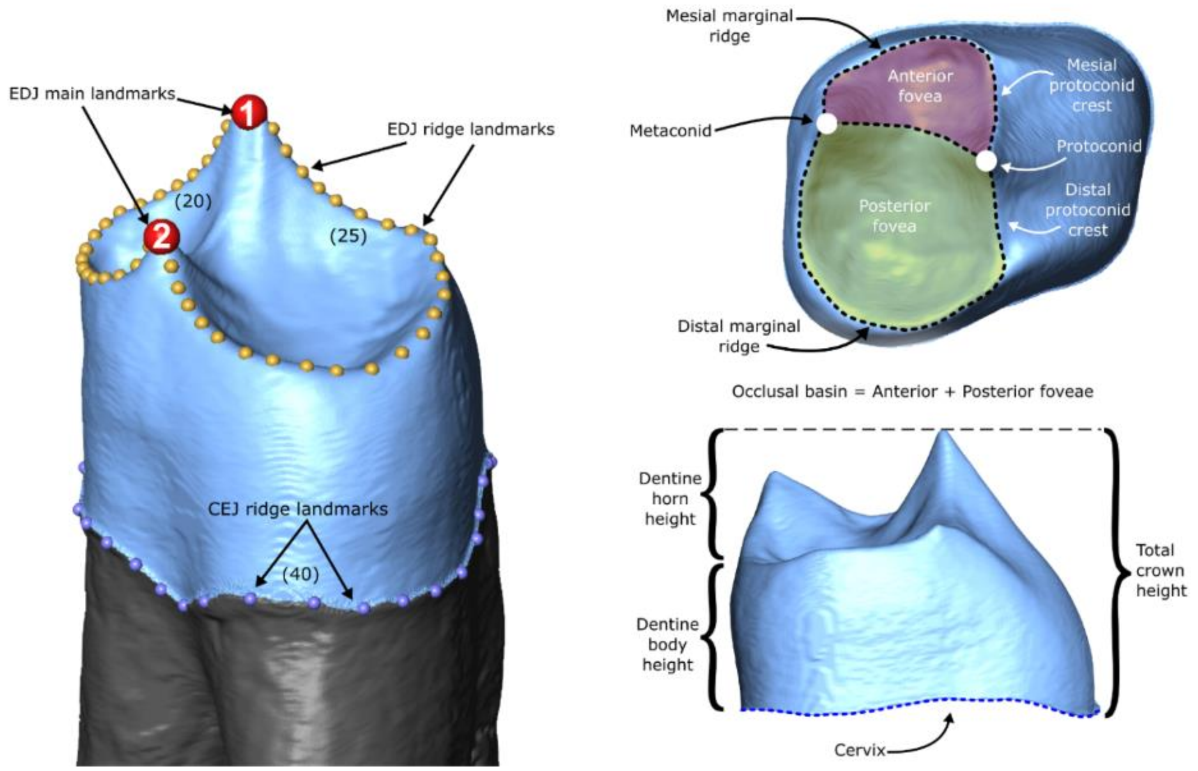

Supplementary Figure 2. Landmarking protocol and terminology guide. Left: Example of the landmarking protocol for the three landmark sets. Numbers in brackets indicate the number of landmarks placed in each set, with the EDJ ridge set split into two sections. 1 = Protoconid landmark, 2 = Metaconid landmark (or homologous point – see text). Right: *H. naledi* right mandibular fourth premolars in occlusal view (top) and mesial view (bottom), illustrating the major morphological features present in hominoid premolars. EDJ images were created in Avizo 6.3 (www.vsg3D.com)

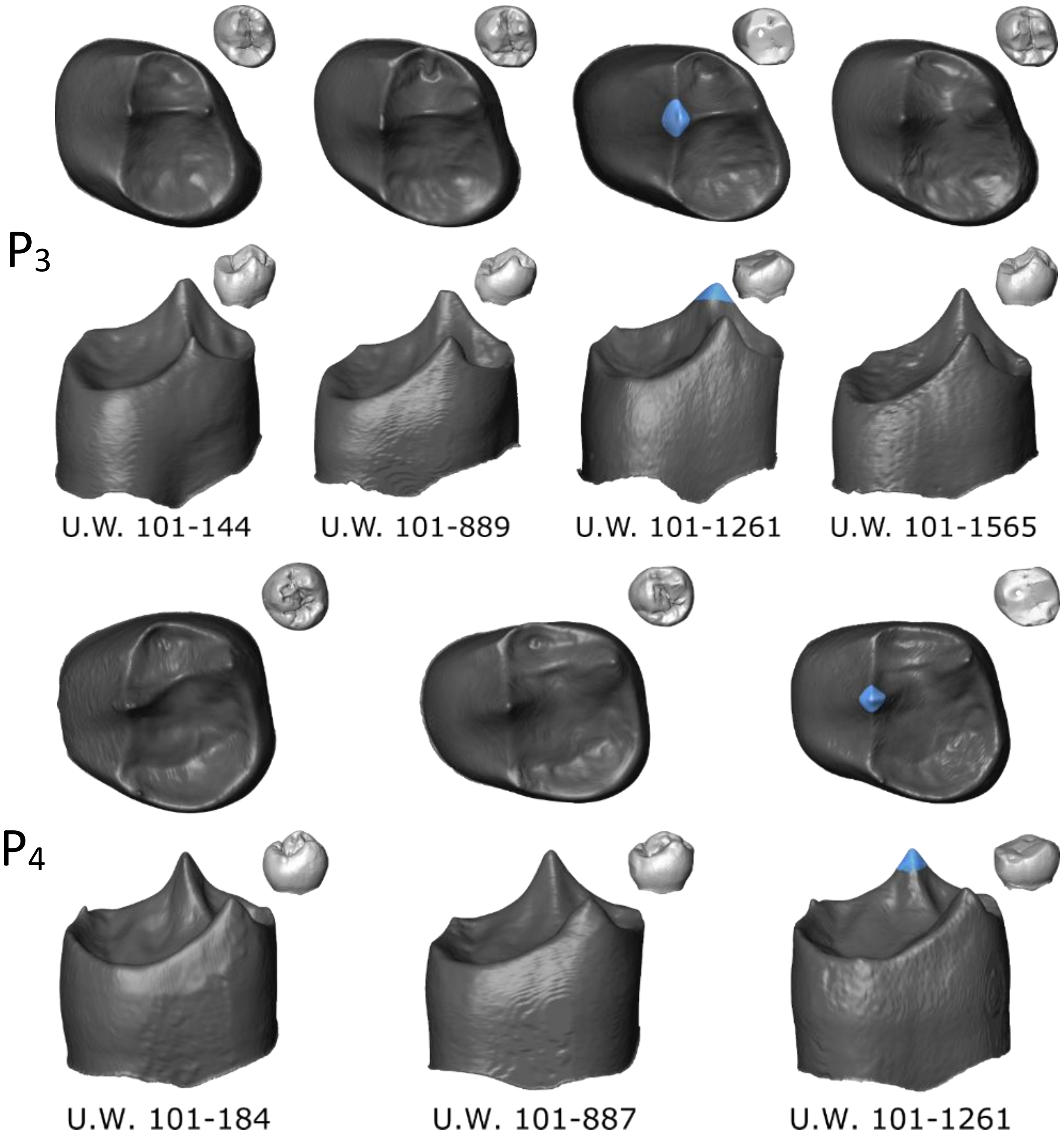

Supplementary Figure 3. Enamel dentine junction morphology of *Homo naledi* premolars. All *H. naledi* premolars included in the *complete* analysis are shown in occlusal (top) and lingual (bottom) view; the outer enamel surface for each specimen is also shown in small. For specimens where the dentine horns have been reconstructed due to wear, the reconstructed sections are shown in blue. EDJ images were created in Avizo 6.3 (www.vsg3D.com)

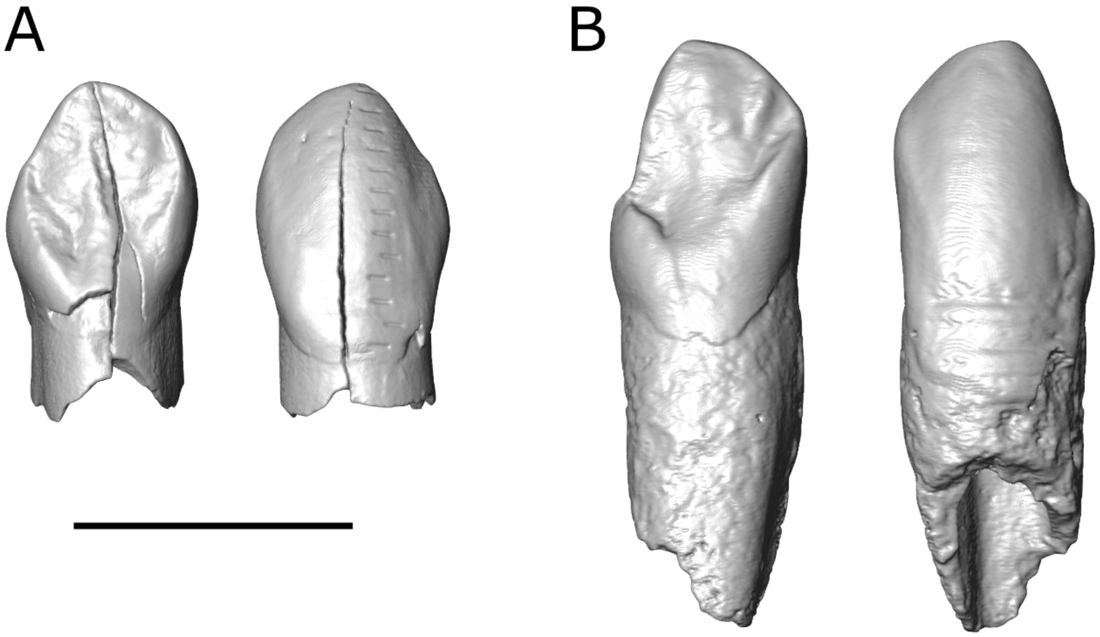

Supplementary Figure 4. Canine morphology in A) SK 96 and B) U.W. 101-985 (*Homo naledi*). For each, the left image is in lingual view and the right in buccal view. Scale bar = 1cm. Isosuface images were created in Avizo 6.3 (www.vsg3D.com)

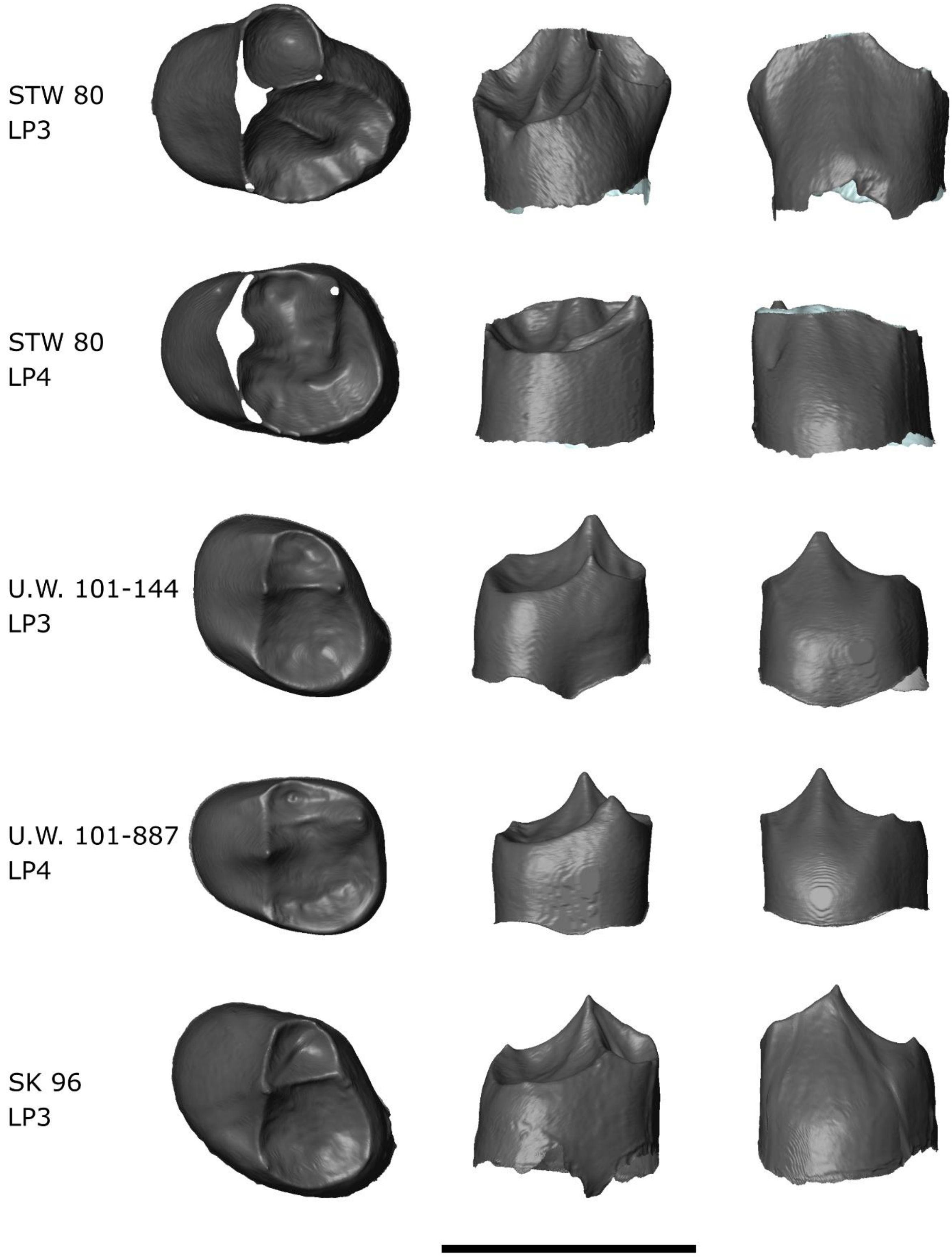

Supplementary Figure 5a. Premolar EDJ morphology in select South African hominins. U.W. 101-144 and U.W. 101-877 represent *Homo naledi*, while STW 80 is assigned to *Homo* sp. and SK 96 was previously attributed to *P. robustus*. For each specimen, EDJ is shown in occlusal (left), lingual (middle) and buccal (right) views. Scale bar = 1cm. EDJ images were created in Avizo 6.3 (www.vsg3D.com)

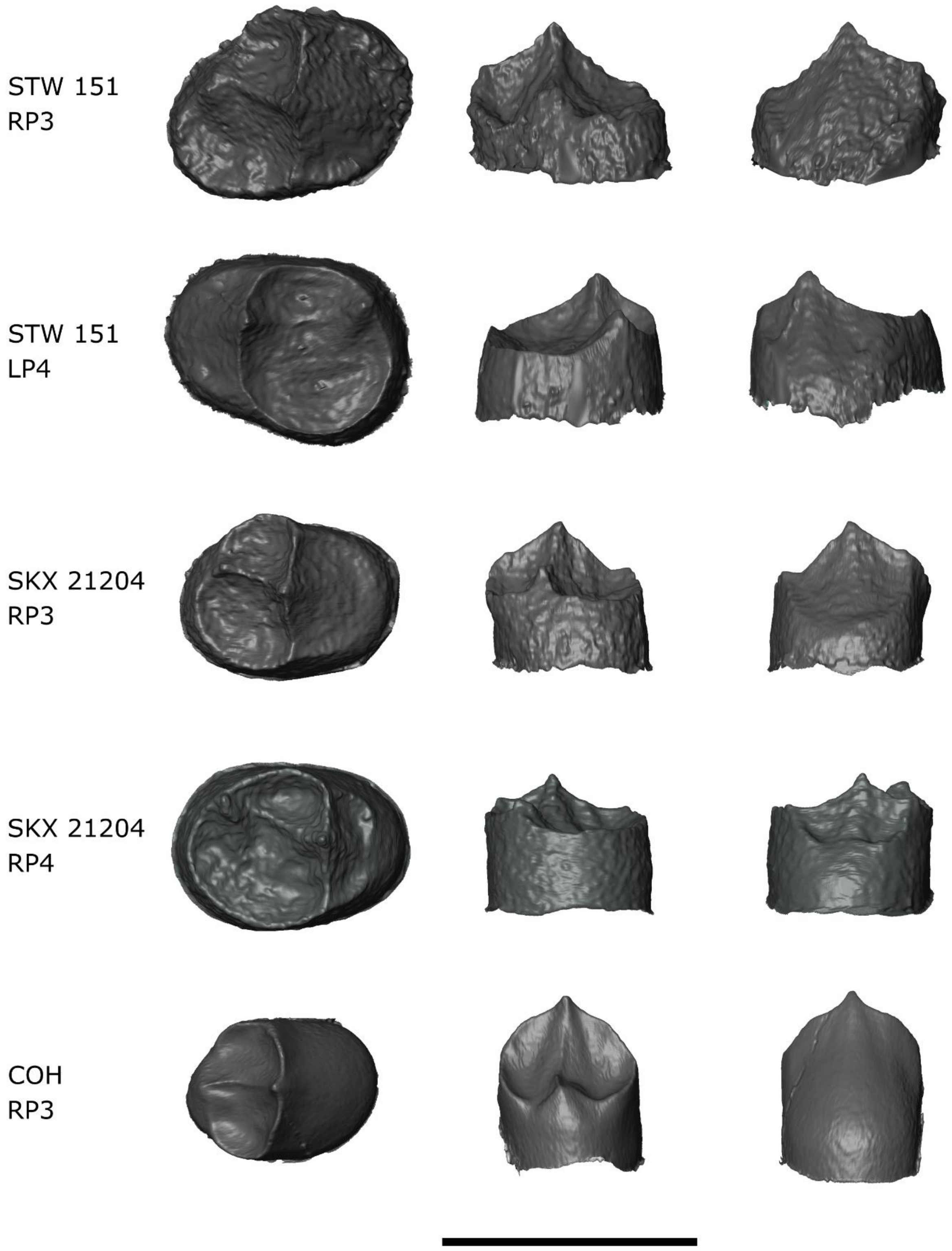

Supplementary Figure 5b. Premolar EDJ morphology in select South African hominins. SKX 21204 is assigned to *Homo* sp., while STW 151 and Cave of Hearths are considered indeterminate . For each specimen, EDJ is shown in occlusal (left), lingual (middle) and buccal (right) views. Scale bar = 1cm. Abbreviations: COH = Cave of Hearths. EDJ images were created in Avizo 6.3 (www.vsg3D.com)

Supplementary Table 1 – Detailed study sample, including which analyses each specimen is included in

Specimen	Tooth	Side	Site/Origin	Taxonomy	Source	Position basis[a]	Position source	*Complete*	*Worn*	Recon ?	Ln(CS)
STS 51	P3	R	Sterkfontein, South Africa	*Australopithecus africanus*	Brain, 1981[1]	2	Brain, 1981[1]	Y	Y	-	3.526
STS 52b	P3	R	Sterkfontein, South Africa	*Australopithecus africanus*	Dart, 1954[2]	1	Dart, 1954[2]	Y	Y	Prd	3.565
STW 7	P3	L	Sterkfontein, South Africa	*Australopithecus africanus*	Moggi-Cecchi et al., 2006[3]	1	Moggi-Cecchi et al., 2006[3]	Y	Y	Prd	3.525
STW 213	P3	R	Sterkfontein, South Africa	*Australopithecus africanus*	Moggi-Cecchi et al., 2006[3]	3	Moggi-Cecchi et al., 2006[3]	Y	Y	Prd	3.403
STW 404	P3	R	Sterkfontein, South Africa	*Australopithecus africanus*	Moggi-Cecchi et al., 2006[3]	2	Moggi-Cecchi et al., 2006[3]	Y	Y	Prd	3.489
DNH8	P3	L	Drimolen, South Africa	*Paranthropus robustus*	Moggi-Cecchi et al., 2010[4]	1	Moggi-Cecchi et al., 2010[4]	Y	Y	-	3.590
DNH46	P3	R	Drimolen, South Africa	*Paranthropus robustus*	Moggi-Cecchi et al., 2010[4]	1	Moggi-Cecchi et al., 2010[4]	Y	Y	-	3.526
DNH51	P3	R	Drimolen, South Africa	*Paranthropus robustus*	Moggi-Cecchi et al., 2010[4]	1	Moggi-Cecchi et al., 2010[4]	N	Y	-	3.543
DNH68	P3	R	Drimolen, South Africa	*Paranthropus robustus*	Moggi-Cecchi et al., 2010[4]	2	Moggi-Cecchi et al., 2010[4]	N	Y	-	3.621
SK23	P3	L	Swartkrans, South Africa	*Paranthropus robustus*	Broom and Robinson, 1952[5]	1	Oakley, 1977[6]	N	Y	-	3.528
SK30	P3	L	Swartkrans, South Africa	*Paranthropus robustus*	Robinson, 1956[7]	3	Oakley, 1977[6]	N	Y	-	3.554
SK100	P3	R	Swartkrans, South Africa	*Paranthropus robustus*	Robinson, 1956[7]	3	Oakley, 1977[6]	Y	Y	-	3.607
SK857	P3	R	Swartkrans, South Africa	*Paranthropus robustus*	Robinson, 1956[7]	3	Oakley, 1977[6]	Y	Y	-	3.609
SKW5	P3	R	Swartkrans, South Africa	*Paranthropus robustus*	Grine and Daegling, 1993[8]	1	Grine and Daegling, 1993[8]	Y	Y	Prd	3.514
KNM-ER 992A	P3	R	Koobi Fora, Kenya	*Homo* aff. *H. erectus*	Wood, 1991[9]	1	Wood, 1991[9]	Y	Y	Prd	3.587
KNM-ER 1507	P3	L	Koobi Fora, Kenya	*Homo* aff. *H. erectus*	Wood, 1991[9]	1	Wood, 1991[9]	Y	Y	-	3.563
KNM-ER 1802	P3	R	Koobi Fora, Kenya	*Homo* sp.	Wood, 1991[9]	1	Wood, 1991[9]	Y	Y	-	3.616
OH7	P3	R	Olduvai Gorge, Tanzania	*Homo habilis*	Leakey et al., 1964[10]	1	Tobias, 1991[11]	Y	Y	-	3.463
OH13	P3	R	Olduvai Gorge, Tanzania	*Homo habilis*	Leakey et al., 1964[10]	1	Tobias, 1991[11]	Y	Y	-	3.419
SK96	P3	L	Swartkrans, South Africa	Indet (*Paranthropus robustus*)	Robinson, 1956[7]	1	Robinson, 1956[7]	Y	Y	Cvx	3.467
SKX 21204	P3	R	Swartkrans, South Africa	*Homo* sp.	Grine, 1989[12]	1	Grine, 1989[12]	Y	Y	-	3.349
STW 151	P3	R	Sterkfontein, South Africa	Indet (*A. africanus*)	Moggi-Cecchi et al., 1998[13]	1	Moggi-Cecchi et al., 1998[13]	Y	Y	-	3.475
STW 80	P3	L	Sterkfontein, South Africa	*Homo* sp.	Moggi-Cecchi et al., 2006[3]	1	Moggi-Cecchi et al., 2006[3]	N	Y	-	3.547
U.W. 101-001	P3	R	Rising Star, South Africa	*Homo naledi*	Berger et al. 2015[14]	1	Berger et al. 2015[14]	N	Y	-	3.458
U.W. 101-0144	P3	L	Rising Star, South Africa	*Homo naledi*	Berger et al. 2015[14]	3	Berger et al. 2015[14]	Y	Y	-	3.397
U.W. 101-0889	P3	L	Rising Star, South Africa	*Homo naledi*	Berger et al. 2015[14]	3	Berger et al. 2015[14]	Y	Y	-	3.384
U.W. 101-1261	P3	R	Rising Star, South Africa	*Homo naledi*	Berger et al. 2015[14]	1	Berger et al. 2015[14]	Y	Y	Prd	3.402
U.W. 101-1565	P3	L	Rising Star, South Africa	*Homo naledi*	Berger et al. 2015[14]	1	Berger et al. 2015[14]	Y	Y	-	3.421
U.W. 101-850	P3	R	Rising Star, South Africa	*Homo naledi*	Berger et al. 2015[14]	3	Berger et al. 2015[14]	N	Y	-	3.451
U.W. 102-0023	P3	R	Rising Star, South Africa	*Homo naledi*	Hawks et al. 2017[15]	2	Hawks et al. 2017[15]	N	Y	-	3.465

Cave of Hearths	P3	R	Cave of Hearths	Indet	Tobias, 1971[16]	1	Tobias, 1971[16]	Y	Y	-	3.288
Combe-Grenal I	P3	R	Combe Grenal, France	*Homo neanderthalensis*	Garralda and Vandermeersch, 2000[17]	1	Garralda and Vandermeersch, 2000[17]	Y	Y	-	3.475
KRP 51	P3	R	Krapina, Croatia	*Homo neanderthalensis*	Radovčić, 1988[18]	1	Radovčić, 1988[18]	Y	Y	-	3.371
KRP 52	P3	L	Krapina, Croatia	*Homo neanderthalensis*	Radovčić, 1988[18]	1	Radovčić, 1988[18]	Y	Y	-	3.334
KRP 54	P3	L	Krapina, Croatia	*Homo neanderthalensis*	Radovčić, 1988[18]	1	Radovčić, 1988[18]	Y	Y	-	3.328
KRP 55	P3	L	Krapina, Croatia	*Homo neanderthalensis*	Radovčić, 1988[18]	1	Radovčić, 1988[18]	Y	Y	-	3.408
KRP D111	P3	L	Krapina, Croatia	*Homo neanderthalensis*	Radovčić, 1988[18]	3	Radovčić, 1988[18]	Y	Y	-	3.500
KRP D114	P3	L	Krapina, Croatia	*Homo neanderthalensis*	Radovčić, 1988[18]	2	Radovčić, 1988[18]	Y	Y	-	3.413
KRP D33	P3	L	Krapina, Croatia	*Homo neanderthalensis*	Radovčić, 1988[18]	2	Radovčić, 1988[18]	Y	Y	-	3.431
KRP D34	P3	R	Krapina, Croatia	*Homo neanderthalensis*	Radovčić, 1988[18]	3	Radovčić, 1988[18]	Y	Y	Prd	3.427
SCLA 4A 6	P3	R	Scladina, Belgium	*Homo neanderthalensis*	Toussaint et al., 1998[19]	2	Toussaint et al., 1998[19]	Y	Y	-	3.353
MPI M139	P3	R	Anatomical collection	*Homo sapiens*	MPI records	3	MPI records	Y	Y		3.338
MPI M34	P3	L	Anatomical collection	*Homo sapiens*	MPI records	3	MPI records	Y	Y	-	3.267
MPI M35	P3	R	Anatomical collection	*Homo sapiens*	MPI records	3	MPI records	Y	Y	-	3.250
ULAC 1	P3	R	Anatomical collection	*Homo sapiens*	ULAC records	1	ULAC records	Y	Y	-	3.227
ULAC 13	P3	L	Anatomical collection	*Homo sapiens*	ULAC records	1	ULAC records	Y	Y	-	3.251
ULAC 536	P3	R	Anatomical collection	*Homo sapiens*	ULAC records	1	ULAC records	Y	Y	Prd	3.144
ULAC 58	P3	L	Anatomical collection	*Homo sapiens*	ULAC records	1	ULAC records	Y	Y	-	3.238
ULAC 790	P3	L	Anatomical collection	*Homo sapiens*	ULAC records	1	ULAC records	Y	Y	-	3.196
ULAC 797	P3	R	Anatomical collection	*Homo sapiens*	ULAC records	1	ULAC records	Y	Y	-	3.255
ULAC 801	P3	L	Anatomical collection	*Homo sapiens*	ULAC records	1	ULAC records	Y	Y	-	3.344
ULAC 806	P3	L	Anatomical collection	*Homo sapiens*	ULAC records	1	ULAC records	Y	Y	-	3.247
STS52B	P4	R	Sterkfontein, South Africa	*Australopithecus africanus*	Brain, 1981[1]	1	Brain, 1981[1]	Y	Y	-	3.615
STW 14	P4	L	Sterkfontein, South Africa	*Australopithecus africanus*	Moggi-Cecchi et al., 2006[3]	1	Moggi-Cecchi et al., 2006[3]	Y	Y	-	3.598
STW131	P4	L	Sterkfontein, South Africa	*Australopithecus africanus*	Moggi-Cecchi et al., 2006[3]	2	Moggi-Cecchi et al., 2006[3]	Y	Y	-	3.649
STW213	P4	R	Sterkfontein, South Africa	*Australopithecus africanus*	Moggi-Cecchi et al., 2006[3]	2	Moggi-Cecchi et al., 2006[3]	Y	Y	Prd	3.546
STW327	P4	L	Sterkfontein, South Africa	*Australopithecus africanus*	Moggi-Cecchi et al., 2006[3]	1	Moggi-Cecchi et al., 2006[3]	Y	Y	-	3.682
STW404	P4	R	Sterkfontein, South Africa	*Australopithecus africanus*	Moggi-Cecchi et al., 2006[3]	1	Moggi-Cecchi et al., 2006[3]	Y	Y	-	3.568
STW537	P4	L	Sterkfontein, South Africa	*Australopithecus africanus*	Moggi-Cecchi et al., 2006[3]	2	Moggi-Cecchi et al., 2006[3]	Y	Y	-	3.739
TM1523	P4	L	Sterkfontein, South Africa	*Australopithecus africanus*	Robinson, 1956[7]	3	Robinson, 1956[7]	Y	Y	-	3.461
SK7	P4	R	Swartkrans, South Africa	*Paranthropus robustus*	Robinson, 1956[7]	3	Oakley, 1977[6]	Y	Y	-	3.702
SK23	P4	R	Swartkrans, South Africa	*Paranthropus robustus*	Broom and Robinson, 1952[5]	1	Oakley, 1977[6]	Y	Y	Prd	3.687
SK55B (SK1589)	P4	L	Swartkrans, South Africa	*Paranthropus robustus*	Brain, 1970[20]	3	Ditsong Records	Y	Y	-	3.709
SK88	P4	L	Swartkrans, South Africa	*Paranthropus robustus*	Robinson, 1956[7]	3	Oakley, 1977[6]	Y	Y	-	3.820

SK826B	P4	L	Swartkrans, South Africa	*Paranthropus robustus*	Robinson, 1956[7]	3	Oakley, 1977[6]	Y	Y	-	3.768
SK1587a	P4	L	Swartkrans, South Africa	*Paranthropus robustus*	Brain, 1970[20]	1	Brain, 1970[20]	Y	Y	Prd	3.637
SK1588	P4	R	Swartkrans, South Africa	*Paranthropus robustus*	Brain, 1970[20]	1	Brain, 1970[20]	Y	Y	-	3.660
SKW5	P4	R	Swartkrans, South Africa	*Paranthropus robustus*	Grine and Daegling, 1993[8]	1	Grine and Daegling, 1993[8]	Y	Y	-	3.664
KNMER 992A	P4	R	Koobi Fora, Kenya	*Homo* aff. *H. erectus*	Wood, 1991[9]	1	Wood, 1991[9]	Y	Y		3.585
KNM-ER 1507	P4	L	Koobi Fora, Kenya	*Homo* aff. *H. erectus*	Wood, 1991[9]	1	Wood, 1991[9]	Y	Y	-	3.554
KNM-ER 1802	P4	R	Koobi Fora, Kenya	*Homo* sp.	Wood, 1991[9]	1	Wood, 1991[9]	Y	Y	Prd	3.712
SKX 21204	P4	R	Swartkrans, South Africa	*Homo* sp.	Grine, 1989[12]	1	Grine, 1989[12]	Y	Y	-	3.438
STW 151	P4	L	Sterkfontein, South Africa	Indet (*A. africanus*)	Moggi-Cecchi et al., 1998[13]	1	Moggi-Cecchi et al., 1998[13]	Y	Y	-	3.483
STW 80	P4	L	Sterkfontein, South Africa	*Homo* sp.	Moggi-Cecchi et al., 2006[3]	1	Moggi-Cecchi et al., 2006[3]	N	Y	-	3.519
OH7	P4	R	Olduvai Gorge, Tanzania	*Homo habilis*	Leakey et al., 1964[10]	1	Tobias, 1991[11]	Y	Y	-	3.555
U.W. 101-0184	P4	L	Rising Star, South Africa	*Homo naledi*	Berger et al. 2015[14]	3	Berger et al. 2015[14]	Y	Y	-	3.366
U.W. 101-0887	P4	L	Rising Star, South Africa	*Homo naledi*	Berger et al. 2015[14]	3	Berger et al. 2015[14]	Y	Y	-	3.350
U.W. 101-1261	P4	L	Rising Star, South Africa	*Homo naledi*	Berger et al. 2015[14]	1	Berger et al. 2015[14]	Y	Y	Prd	3.386
U.W. 102-0023	P4	R	Rising Star, South Africa	*Homo naledi*	Hawks et al. 2017[15]	2	Hawks et al. 2017[15]	N	Y	-	3.428
Combe Grenal 29	P4	L	Combe Grenal, France	*Homo neanderthalensis*	Garralda and Vandermeersch, 2000[17]	3	Garralda and Vandermeersch, 2000[17]	Y	Y	-	3.394
Combe Grenal IV	P4	L	Combe Grenal, France	*Homo neanderthalensis*	Garralda and Vandermeersch, 2000[17]	1	Garralda and Vandermeersch, 2000[17]	Y	Y	-	3.462
Combe Grenal VIII	P4	L	Combe Grenal, France	*Homo neanderthalensis*	Garralda and Vandermeersch, 2000[17]	3	Garralda and Vandermeersch, 2000[17]	Y	Y	Prd, Med	3.372
KRP 52	P4	L	Krapina, Croatia	*Homo neanderthalensis*	Radovčić, 1988[18]	1	Radovčić, 1988[18]	Y	Y	-	3.386
KRP 58	P4	R	Krapina, Croatia	*Homo neanderthalensis*	Radovčić, 1988[18]	1	Radovčić, 1988[18]	Y	Y	Prd	3.435
KRP D35	P4	R	Krapina, Croatia	*Homo neanderthalensis*	Radovčić, 1988[18]	3	Radovčić, 1988[18]	Y	Y	-	3.503
KRP D50	P4	L	Krapina, Croatia	*Homo neanderthalensis*	Radovčić, 1988[18]	3	Radovčić, 1988[18]	Y	Y	-	3.459
Regourdou 1	P4	L	Le Regourdou, France	*Homo neanderthalensis*	Maureille et al., 2001[21]	1	Maureille et al., 2001[21]	Y	Y	-	3.296
MPI M14	P4	L	Anatomical collection	*Homo sapiens*	MPI records	3	MPI records	Y	Y	-	3.320
ULAC 1	P4	R	Anatomical collection	*Homo sapiens*	ULAC records	1	ULAC records	Y	Y	-	3.245
ULAC 13	P4	L	Anatomical collection	*Homo sapiens*	ULAC records	1	ULAC records	Y	Y	-	3.286
ULAC 171	P4	L	Anatomical collection	*Homo sapiens*	ULAC records	1	ULAC records	Y	Y	Prd	3.271
ULAC 179	P4	R	Anatomical collection	*Homo sapiens*	ULAC records	1	ULAC records	Y	Y	Prd	3.288
ULAC 536	P4	R	Anatomical collection	*Homo sapiens*	ULAC records	1	ULAC records	Y	Y	-	3.204
ULAC 58	P4	L	Anatomical collection	*Homo sapiens*	ULAC records	1	ULAC records	Y	Y	-	3.298
ULAC 607	P4	R	Anatomical collection	*Homo sapiens*	ULAC records	1	ULAC records	Y	Y	Prd	3.378
ULAC 790	P4	L	Anatomical collection	*Homo sapiens*	ULAC records	1	ULAC records	Y	Y	-	3.265
ULAC 797	P4	R	Anatomical collection	*Homo sapiens*	ULAC records	1	ULAC records	Y	Y	-	3.339

Abbreviations: EDJ+CEJ = analysis using all landmark sets; Lesedi = analysis using only landmarks corresponding to sections preserved in LES1 premolars; ln(CS) = natural logarithm of centroid size (calculated from the Lesedi analysis); Recon? = Specimens with reconstructed dentine horns (Prd = protoconid reconstructed; Med = metaconid reconstructed)
[a]Position basis; 1 = In jaw, 2 = Associated dentition, 3 = based on morphology

References

1. Brain, C.K. *The hunters or the hunted?: an introduction to African cave taphonomy.* (University of Chicago Press, 1981).
2. Dart, R.A. The second, or adult, female mandible of *Australopithecus prometheus*. *Am. J. Phys. Anth.* **12,** 313-344. (1954).
3. Moggi-Cecchi, J., Grine, F. E. & Tobias, P. V. Early hominid dental remains from Members 4 and 5 of the Sterkfontein Formation (1966–1996 excavations): catalogue, individual associations, morphological descriptions and initial metrical analysis. *J. Hum. Evol*. **50,** 239-328 (2006).
4. Moggi-Cecchi, J., Menter, C., Boccone, S. & Keyser, A. Early hominin dental remains from the Plio-Pleistocene site of Drimolen, South Africa. *J. Hum. Evol*. **58,** 374-405 (2010).
5. Broom, R., and Robinson, J.T. *Swartkrans ape-man. Paranthropus crassidens* (Transvaal Museum, 1952).
6. Oakley, K., Campbell, B., and Molleson, T. *Catalogue of fossil hominids part 1: Africa*. (British Museum (Natural History), 1977).
7. Robinson, J. T. *The Dentition of Australopithecinae*. (Transvaal Museum, 1956).
8. Grine F.E. and Daegling D.J. New mandible of *Paranthropus robustus* from Member 1, Swartkrans Formation, South Africa. *J. Hum. Evol.* **24,** 319-333 (1993).
9. Wood, B. A. *Koobi Fora Research Project: Volume 4. Hominid cranial remains* (Clarendon Press, 1991).
10. Leakey, L.S.B., Tobias, P.V., Napier, J.R. A new species of the genus *Homo* from Olduvai Gorge. Nature **202,** 7-9 (1964).
11. Tobias, P. V. *The Skulls, Endocasts, and Teeth of Homo habilis* (Cambridge University Press, 1991).
12. Grine, F. E. New hominid fossils from the Swartkrans formation (1979-1986 excavations): craniodental specimens. *Am. J. Phys. Anth.* **79,** 409-449 (1989).
13. Moggi-Cecchi, J., Tobias, P. V. & Beynon, A. The mixed dentition and associated skull fragments of a juvenile fossil hominid from Sterkfontein, South Africa. *Am. J. Phys. Anth.* **106,** 425-465 (1998).
14. Berger, L. R. *et al. Homo naledi*, a new species of the genus *Homo* from the Dinaledi Chamber, South Africa. *eLife* **4,** e09560 (2015).
15. Hawks, J. *et al.* New fossil remains of *Homo naledi* from the Lesedi Chamber, South Africa. *Elife* **6,** e24232 (2017).
16. Tobias, P.V. Human skeletal remains from the Cave of Hearths, Makapansgat, northern Transvaal. *Am. J. Phys. Anth.* **34,** 335-367. (1971).
17. Garralda, M.D. and Vandermeersch, B. Les Néandertaliens de la grotte de Combe-Grenal (Domme, Dordogne, France). *Paléo* **12,** 213-259 (2000).
18. Radovčić, J., *The Krapina hominids: an illustrated catalog of skeletal collection* (Mladost, 1988).
19. Toussaint, M. *et al.* Les restes humains néandertaliens immatures de la couche 4A de la grotte Scladina (Andenne, Belgique). *C. R. Acad. Sci.* **326,** 737-742 (1998).
20. Brain C.K. New finds at the Swartkrans australopithecine site. *Nature* **225,** 1112-1119 (1970).
21. Maureille, B., Rougier, H., Houët, F. and Vandermeersch, B. Les dents inférieures du Néandertalien Regourdou 1 (site de Regourdou, commune de Montignac, Dordogne): Analyses métriques et comparatives. *PALEO* **13,** 183-200 (2001).
